# ‘It Simply Required Far Too Many Steps and Made You Feel You Were Just a Number’. Family Caregivers' Experiences With Assisted Suicide in Austria: A Qualitative Study

**DOI:** 10.1111/hex.70651

**Published:** 2026-03-27

**Authors:** Tamina‐Laetitia Vielgrader, Ricarda Mewes, Julia Fischer, Jana Marica Hluch, Maria Kletecka‐Pulker, Gudrun Kreye, Elisabeth Lucia Zeilinger

**Affiliations:** ^1^ Institute for Ethics and Law in Medicine University of Vienna and Medical University of Vienna Vienna Austria; ^2^ Vienna Doctoral School in Cognition, Behaviour and Neuroscience University of Vienna Vienna Austria; ^3^ Faculty of Social Sciences Institute of Psychology, University of Klagenfurt Klagenfurt Austria; ^4^ Ludwig Boltzmann Institute Digital Health and Patient Safety Ludwig Boltzmann Gesellschaft Vienna Austria; ^5^ Division of Palliative Care, Division of Internal Medicine 2 University Hospital Krems Krems Austria; ^6^ Karl Landsteiner University of Health Sciences Krems Austria; ^7^ Department of Clinical and Health Psychology, Faculty of Psychology University of Vienna Vienna Austria; ^8^ Department of Clinical Research SBG Academy for Ageing Research, Haus der Barmherzigkeit Vienna Austria; ^9^ Division of Health Psychology, Faculty of Psychology Karl Landsteiner University of Health Sciences Krems Austria

**Keywords:** assisted dying, assisted dying regulation, assisted suicide, family caregivers, qualitative research, semi‐structured interviews, thematic analysis

## Abstract

**Introduction:**

This study examines the lived experiences of family caregivers navigating the legal and emotional complexities of Austria's newly enacted Dying Decree Law.

**Methods:**

A qualitative interview study was conducted with nine family caregivers (eight women) of people seeking assisted suicide (PSAS) in Austria. Participants were recruited through purposive sampling through multiple recruitment channels. Interviews were analysed using thematic analysis (supported by MAXQDA).

**Results:**

Seven overarching themes emerged: (1) Dynamics of Dying and Saying Goodbye; (2) Inclusion versus Exclusion in the Decision‐Making Process; (3) Role Negotiation and Responsibilities; (4) Bureaucratic and Legal Barriers; (5) Social Exclusion and Stigmatisation; (6) Support Needs and Structures; and (7) Ramifications in the Post‐Mortem Phase. Family caregivers reported a lack of formal guidance, often leading to isolation and exhaustion. Open communication was repeatedly highlighted as essential in order to honour the PSAS's wishes and alleviate family caregivers' grief.

**Conclusion:**

Family caregivers function as advocates and bureaucratic managers while simultaneously serving as carers who provide the PSAS with physical and emotional support. Rather than relying on the legal framework alone, public policy should also formally acknowledge this dual role and deliver government‑led structural support for family caregivers to lessen their emotional and bureaucratic burden.

**Patient or Public Contribution:**

Family caregivers of PSAS were central to this research. We interviewed family caregivers about their experiences with assisted dying under Austria's Dying Decree Law, and incorporated their suggestions for addressing the identified challenges and improving support structures. Their contributions were integral to the study's findings and its focus on enhancing support structures for family caregivers within assisted dying systems.

**Abbreviations**
AS: assisted suicide
PSAS: people seeking assisted suicide

## 1 Introduction

Across the world, assisted dying laws are being introduced and are evolving, yet families and friends remain the hidden stakeholders in the process [1, 2, 3, 4, 5, 6]. International research has already shown that family caregivers are not specifically named in legal frameworks, despite occupying a distinct role that goes beyond providing care and assistance. Their involvement in the assisted suicide (AS) process is therefore of the utmost importance, yet it is often underestimated. As part of the AS process, relatives or friends often find themselves responsible for researching legal and medical requirements, organising procedural steps, and offering emotional and practical support [2, 7, 8]. Even though legislation differs between countries which allow assisted dying, several studies conducted in the Netherlands, Canada, and Australia have shown that there is a common theme with respect to acquiring knowledge and information: where no physician or institution is responsible for guiding them and the people seeking assisted suicide (PSAS) through the process, family caregivers are often left to navigate a bureaucratic maze on their own [9, 10]. Several studies have shown that family caregivers who felt unsupported in these situations reported an increased emotional burden as they tried to navigate their role [2, 3, 11]. In Austria, the Dying Decree Law came into effect on January 1, 2022. The legislator decided that Austria would permit AS as a form of assisted dying, with the PSAS needing to establish a dying decree, that is, a legal document recording their free and self‐determined will and intention to die by AS. The legal framework defines the step‐by‐step and time‐intensive process the PSAS must follow. It begins with meeting the eligibility criteria and undergoing separate mandatory consultations with two physicians, one of whom must hold a special qualification in palliative medicine. After these consultations, a dying decree is issued by a notary or a patient advocate. This document authorises the individual to obtain the lethal preparation (sodium pentobarbital), which can then be dispensed by a public pharmacy. While the law does not explicitly specify relatives, it does allow the PSAS to name ‘assisting persons’ in the dying decree—trusted individuals who may offer support with the practical aspects of the process, such as collecting the preparation from the pharmacy.

To date, there is no empirical research examining how relatives view or experience Austria's Dying Decree Law, highlighting the lack of qualitative data in newly legalised assisted suicide contexts. Furthermore, it is unclear whether international research findings can be applied to Austria, where the law is new and structural guidance is absent. Therefore, this is the first Austrian study on bereaved family members of PSAS. Our study aims to examine family caregivers' experiences across the entire AS process in Austria, including decision‐making, bureaucratic procedures, role negotiation, social dynamics, support structures and the post‐mortem phase. Our research adopts an emancipatory perspective, which prioritises giving voice to participants and highlighting their lived experiences, particularly in contexts where they may be overlooked or marginalised. This perspective guided our methodology and analysis by ensuring that participants' accounts were interpreted in ways that respect their agency and perspectives. During analysis, coding and theme development focused on capturing participants' perspectives authentically, emphasising challenges, needs and strategies identified by family caregivers themselves.

International research has consistently shown that family caregivers often lack political awareness of their own concerns [12, 13]. In addition, they frequently perceive caregiving as their personal responsibility and a private matter. However, one could argue that what they fail to recognise is that what they perceive as their personal engagement is actually the result of gaps in the healthcare system, which assigns family caregivers the role of unpaid service providers [14, 15]. Existing research on this matter highlights the importance of making the experiences of family caregivers visible in order to understand their contributions to and challenges within the system [16]. In the context of AS, stigmatisation and social exclusion are additional factors, preventing family caregivers from networking and exchanging information as a means of accessing support. As a result, the dialogue has become imbalanced, creating a division between those who speak about AS, and those who are spoken about.

The objective of our study is to contribute to a less stigmatised and more inclusive debate [17]. By elucidating these dimensions, our research aims to inform the development of structured guidance for family caregivers and PSAS, and to provide greater visibility for the experiences and perspectives of those affected by the AS process.

## 2 Methods

The present study comprised nine semi‐structured interviews with bereaved family caregivers who served as confidants to PSAS (within the framework of the Dying Decree Law) in Austria. Our research adopted a phenomenological orientation to data collection and analysis.

### 2.1 Participant Recruitment

To recruit family caregivers of PSAS, we approached physicians practicing in Austria who had declared themselves available for the mandatory consultations needed to establish a dying decree. We contacted the Austrian medical chambers in each of Austria's nine federal states to obtain lists of these physicians providing consultations. We were informed that each federal state's medical chamber maintains its own procedures for handling sensitive data, with the result that physicians were contacted through multiple channels via the respective medical chambers. These included filtering publicly available information on the associations' websites, compiling names and lists and conducting supplementary research to identify contact details, as well as collaborating directly with the medical chambers in Austria to ensure that invitations were forwarded to physicians without compromising their privacy. In addition, the study information sheet was disseminated via medical chamber newsletters, professional magazines and designated website pages. All nine medical chambers in Austria agreed to distribute the study information sheet or provide lists of consulting physicians.

We designed two study information sheets: one for physicians who provided consultations to PSAS and one for family members and confidants. The information sheet for physicians included a request to distribute the attached information sheet to interested family members and confidants. The study information sheet for participants included a detailed description of the study and doctoral project, as well as contact information of the first author, T.L.V. Participant inclusion criteria were as follows: (a) a close relative or confidant of a person who died by AS or wished to die by AS; (b) above the age of 18; (c) able to give informed consent; and (d) with the English or German skills needed to participate in the interview. No formal exclusion criteria were applied to the study population. Given the sensitive nature of the topic and the relatively small number of AS cases in Austria, an inclusive recruitment strategy was adopted to capture the widest possible range of family caregiver perspectives.

### 2.2 Study Participants

Each of the nine interviewees (eight women) represented an individual case; none of the interviewees had met or were related to each other. To protect confidentiality, all participants were pseudonymised. Participants could choose their own pseudonym to facilitate recognition of their quotations in the manuscript. Table 1 shows the participants' demographic data, as well as other contextual information.

**Table 1:** Description of the participants and contextual information.

Pseudonym	Gender	Relation to the PSAS	Occupation	Time of interview
Max	m	Acquaintance	Retired, volunteer	7 months post‐mortem
Beatrice	w	Wife	Working	2 years post‐mortem
Anna	w	Ex‐wife and mother of shared child	Working	1.5 years post‐mortem
Rachel	w	Mother	Working	9 months post‐mortem
Stephanie	w	Daughter	Working	1 year post‐mortem
Giselle	w	Wife	Working	1.5 years post‐mortem
Jane	w	Wife	Retired	1 month pre‐mortem
Naomi	w	Ex‐wife and mother of shared child	Working	3 years post‐mortem
Eloise	w	Wife	Working	2 years post‐mortem

### 2.3 Interview Guide

An interview guide was designed by the multidisciplinary research team (social science, psychology, medical law and medical ethics). Prior to each interview, the participants were contacted via email or telephone and asked to provide key background information about their case (e.g., date of diagnosis, date of death). These details were organised chronologically to inform the development of the interview guide and to enable a more comprehensive exploration of participants' personal stories, backgrounds and experiences. A formal pilot test of the interview guide was not conducted due to the small and hard‐to‐reach population as well as the sensitive nature of the topic.

The interview guide was based on the contents and intended process for PSAS within the Dying Decree Law, starting with the initial wish to die and the mandatory consultations with physicians, to the collection of the lethal preparation (sodium pentobarbital) from the pharmacy, to the occurrence of the death. The interview guide comprised questions covering the involvement in the process and grieving periods, and explored guidance, the social support system, barriers to the process and any adaptations to the current legislation they might wish to see.

### 2.4 Data Collection

The interviews were conducted at a time and place of the participants' choice. The interviews were conducted between November 2024 and September 2025, either in person (*n* = 3) or virtually (*n* = 6). Interviews were conducted until data saturation was achieved, meaning no new themes or insights emerged from additional interviews. Due to the recent legalisation of AS in Austria in 2022, the total number of cases remains low, and publicly available data on occurrences are limited. Saturation was reached after eight interviews, with one additional interview conducted to confirm that no new themes emerged. Interviews ranged from 45 to 120 min and were audio‐recorded after obtaining written and oral informed consent from the participant. All interviews were conducted in German. Audio data were securely transmitted to an external transcription company for verbatim transcription in the original language. All personal data were pseudonymised before analysis.

### 2.5 Data Analysis

We employed thematic analysis as outlined by Braun and Clarke [18]. The analysis followed a systematic six‐step process: (1) familiarisation with the data, (2) generation of initial codes, (3) searching for themes, (4) reviewing potential themes, (5) defining and naming themes and (6) producing the final report. We adopted an inductive approach to maintain flexibility and capture nuanced insights throughout the coding process. After familiarising ourselves with all the data, we developed an initial coding framework that was tested on three interviews. Afterwards, the authors, T.L.V. and J.M.H., coded blindly (without access to each other's coding decisions). The preliminary coding frameworks were subsequently reviewed and adapted to synthesise the codes into themes. MAXQDA was used for data analysis [19]. The authors, T.L.V. and J.M.H., debriefed on a regular basis during data collection and analysis to discuss interpretations, resolve discrepancies and refine the coding framework. These debriefings were also used to explore and articulate potential concerns, ethical considerations, researcher engagement and positionality.

All interviews were analysed in German. Themes were initially developed in German and then translated for the publication. All the following citations were carefully chosen and translated for the purpose of exemplifying and highlighting the issues at hand. Translations were performed by TLV initially and then verified and edited by a professional translator who was also given the German excerpt of the citation. This procedure ensured methodological rigor and conceptual equivalence.

### 2.6 Ethical Considerations

Ethics approval was granted by the University of Vienna Ethics Committee on 11 October 2024, as an addendum to our project ‘Multiperspective evaluation of the Dying Decree Law’ (no 01071). Participants were given the informed consent form 1 week before the scheduled interview to study its contents. Prior to starting the interview, the researcher, T.L.V., again explained the purpose of the study and the information provided in the form before obtaining informed consent from the participants. All transcripts were reviewed before analysis to remove any person‐specific information to ensure pseudonymisation. As the topics discussed were sensitive and possibly triggering for some participants, we decided not to return the transcripts for additional comments or approval.

## 3 Results

**Figure 1:**
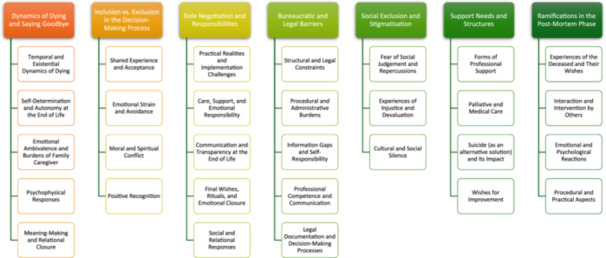
Identified themes and subthemes.

We identified seven themes in the interviewee statements about experiences with the AS process in Austria: (1) Dynamics of Dying and Saying Goodbye; (2) Inclusion versus Exclusion in the Decision‐Making Process; (3) Role Negotiation and Responsibilities; (4) Bureaucratic and Legal Barriers; (5) Social Exclusion and Stigmatisation; (6) Support needs and structures; and (7) Ramifications in the Post‐Mortem Phase. The identified themes have been arranged by chronological order, reflecting the experiences of this period as recounted by interviewees (see Figure 1).

### 3.1 Themes

#### 3.1.1 Dynamics of Dying and Saying Goodbye

Many interviewees experienced a mixture of emotions in the period leading up to the death, including sadness, relief and stress. The majority said that there had often been occasions during which they were annoyed by the legal and medical requirements and practicalities, which they felt stripped them of precious time with their loved one. Interviewees said they often shielded the PSAS by disguising their own anguish in order to appear strong for them. Family caregivers frequently grappled with internal conflicts, questioning whether they had made the right decisions, if the timing was appropriate, or if they could have prolonged life. Despite their lingering doubts, family caregivers found solace in the knowledge that the PSAS no longer had to suffer and that it had been possible to fulfil their wish for a peaceful, self‐determined and dignified death. This solace was often described in terms of ‘liberation’ or ‘relief’.

And in the moment when I saw that he was dead, I felt relieved. You could see it in his face, how much pain he had been in. And I was so glad for him that it was over. And I think it was the same for the boys. We were simply relieved.

(Beatrice, Pos. 251)

Some family caregivers also reflected on the nature of grief, distinguishing between mourning the deceased and their own self‐pity, noting that much of the expressed sorrow can be self‐referential.

Of course, I also miss moments when I would like to be with my husband. Moments when I could simply call him, please, and tell him how the day went or ask for advice. But I differentiate quite clearly: Is this just me feeling sorry for myself again, or is it truly this longing for someone? Most people just feel sorry for themselves. It's simply that, yes.

(Giselle, Pos.58)

Giselle's distinction highlights a significant cognitive strategy used by some caregivers to compartmentalise their emotional pain. By framing certain aspects of grief as ‘self‐pity’, caregivers may attempt to maintain a sense of objective distance from their loss.

Several interviewees also explained that this experience had led to a profound shift in their own perspective, seeing trivial matters as less important and feeling a deeper connection to life and their loved ones.

#### 3.1.2 Inclusion Versus Exclusion in the Decision‐Making Process

We found that the decision‐making process surrounding AS often involves varying degrees of inclusion and exclusion of family and friends. Some PSAS openly discussed their intentions with their nuclear family and close friends, even involving them in planning their funeral and final arrangements, indicating this was a shared journey involving mutual support. Communication within the family was perceived as crucial to ensure everyone is prepared and understands the process and the decisions behind it. Three family caregivers said that their underage children were aware of the AS process. They highlighted the importance of open communication throughout this time to create a safe space in which the children could communicate their feelings openly with the PSAS and the family caregiver.

This open communication was also seen as a way to mitigate the emotional burden while simultaneously fulfilling the PSAS's wishes, even if it meant family caregivers having to overcome personal reservations or religious beliefs. Anna, a bereaved ex‐wife, said:

But I was glad that we took the process so seriously and that we were a part of it. That's life. Life consists of love and dying, and that's just how it is. It was important to me that during this time, the three of us gave each other the feeling that we were there for one another. That was important to me, yes.

(Anna, Pos. 64)

Anna's reflection suggests that for some caregivers, the inclusion in the AS process transforms the act from a medical or legal event into a profound familial milestone. By framing ‘love and dying’ as inseparable components of life, caregivers are able to recontextualise their participation not as a burden, but as a final act of solidarity. This highlights a significant pattern in the data: when families are actively included, the transparency of the process acts as a protective factor against the trauma of the loss.

In contrast, many instances recounted by family caregivers highlighted the deliberate exclusion of family and friends from the decision‐making process, often to spare them distress or because the PSAS preferred to keep their plans private. Some interviewees reported feeling shocked or betrayed when learning about the plans, making the death feel more like a sudden suicide.

In our case, it really felt more like a normal suicide. Because that's also something you don't know anything about and then it suddenly happens. And even though it was very gentle for him, for us it was brutal. […] That was a shock for the parents. Both parents, now approaching 80, are still alive and were deeply affected. And nor did they have the opportunity to say goodbye. And then there's a sister and a brother, and they didn't know anything about it either. Same with friends.

(Naomi, Pos. 104, 40)

#### 3.1.3 Role Negotiation and Responsibilities

Family caregivers frequently assume the role of informal and unpaid carers and coordinators. While some PSAS preferred to manage and organise the AS process themselves, others relied heavily on the support of their family caregivers, including navigating bureaucratic hurdles such as obtaining necessary documents, navigating medical consultations, and dealing with pharmacies.

We found that the role family caregivers inhabit ranges from providing practical assistance and emotional support to managing communication with other professionals. Eloise described how she simultaneously managed multiple roles, including those of wife, mother, nurse and caregiver, while also helping her husband establish a dying decree:

That was, yes, that time was, of course, extremely stressful, especially with the dying decree. That added another layer…There was the caregiving, I have a job, a 14‐year‐old daughter, and I go to work, and then there was the care for my husband. On top of that, I constantly had to organise medications and was always running around. And then this was yet another thing, essentially a huge, huge project that I had to manage relatively alone on the side, with all the medical consultations, appointments, and errands. So, it really was an enormous additional burden.

(Eloise, Pos.58)

Eloise's account illustrates the accumulation of roles, where the dying decree is not merely a document, but a demanding project superimposed onto existing domestic and professional duties. This highlights a significant pattern in our data: the legal bureaucracy of AS in Austria does not exist in a vacuum, but competes for the caregiver's limited time and emotional energy. The significance of this additional burden lies in its potential to marginalise the caregiver's primary role as a supportive family member, forcing them into the role of a bureaucratic manager.

#### 3.1.4 Bureaucratic and Legal Barriers

We identified that the process of establishing a dying decree is highly bureaucratic and challenging, particularly for individuals with no prior knowledge of the process or support. Many family caregivers reported that they had been made aware of the option of AS in Austria through the media or personal connections. Interviewees reported that the process was a bureaucratic maze, involving complex medical and legal requirements. This was perceived as particularly challenging for PSAS, who are constrained by the progressive nature of their terminal or chronic illness.

A recurring theme throughout all the interviews was ‘time’. Family caregivers reported that the process of establishing a dying decree was time‐consuming and that they would rather have spent that time with the PSAS. As there is no standardised procedure involving information centres or appointed support organisations in Austria, weeks were spent researching willing medical professionals for the consultations and notaries. Some interviewees also shared that the search for willing physicians was often serendipitous, with some physicians uncooperative, unprofessional, alternative (homeopathic) or even exploitative in their billing practices.

Especially with that one doctor – after realising that he is very alternative, homeopathic, and a bit convoluted – it left an even more bitter taste in my mouth, I have to say. Also the question of who is even allowed to offer or provide this service in the first place.

(Stephanie, Pos.39)

The majority reported that willing physicians wanted the PSAS to come in for several consultations, not just the two individual consultations required by law. This was not only physically taxing for the PSAS in question, but also financially burdensome and difficult to organise for family caregivers who work full‐time.

The consultation fees range from 120 euros to over 500 euros. I mean, excuse me, what is that all about? The assisting person has work but is not really allowed to charge for it. At least that's what the law says. And the notary can charge whatever they want anyway.

(Giselle, Pos. 156)

Interviewees all shared a strong desire for a streamlined and standardised process that could provide support for family caregivers and the PSAS, ideally managed by a government‐affiliated organisation.

A place where people can turn to, where – whether through freelance contracts or whatever – doctors, notaries, and support providers are employed by the Austrian state, it doesn't matter in what form, and they receive payment for the time they spend, but cannot generate additional income in some other way.(…). And regulated as far as, for example, just like we have counselling centres for expectant mothers, we set up a counselling centre for ‘I want to die’, for assisted suicide, with people employed by the state so that, to put it bluntly, no one can exploit the system.

(Giselle, Pos. 156)

#### 3.1.5 Social Exclusion and Stigmatisation

We found that the process of organising an AS within the framework of the Dying Decree Law was often accompanied by social exclusion and stigmatisation. Several interviewees said that they would not tell friends or family if their loved one intended to die by AS, for fear that they would not understand and would react negatively, spoiling the precious time the PSAS had left. Amongst the caregivers interviewed, this fear was so pervasive that they advised against discussing the matter with anyone who might not understand (mostly attributing misunderstanding to religious attitudes such as Catholic or other Christian beliefs).

We found a consistent feeling of insignificance or depersonalisation among family caregivers and PSAS: their experience in navigating the bureaucratic hurdles involved in planning an AS often left them feeling like anonymous supplicants with no rights. Stephanie, a bereaved daughter, said:

Yes, it simply required far too many steps, and made you feel you were just a number.

(Stephanie, Pos. 82)

Interviewees also shared instances of unprofessional conduct, such as the repeated disclosure of sensitive personal information by administrative staff, which only served to increase the emotional burden on those already undergoing a challenging process.

Some interviewees recounted the struggle to obtain and finally to understand the information provided, highlighting the lack of accessible information for people with lower cognitive abilities or fewer financial resources. Eloise described this period of perceived loneliness and isolation.

It's just really difficult when you go public with this topic; you're met with total anxiety and helplessness, yes. And you just sit there, needing help, but you encounter professionals who are just there and don't really seem able or willing to do anything. So that was really difficult. We were very much left to ourselves. And, well, as a family member – because ultimately I arranged everything – I would have expected support in that situation.

(Eloise, Pos. 28)

Eloise's account underscores that the ‘struggle’ for information is not merely a logistical hurdle but a psychological burden. Furthermore, her critique of professionals who were ‘just there’ but ‘not able or willing’ illustrates a form of institutional abandonment. This highlights that for families without specialised knowledge or resources, the responsibility of arranging the AS process independently leads to a state of isolation. Moreover, a feeling of powerlessness and having to struggle were a persistent theme in the majority of the interviews.

#### 3.1.6 Support Needs and Structures

Despite the various experiences and fears of social exclusion and stigmatisation, interviewees reported having a few trusted confidants who supported them. They highlighted the importance of this ‘inner circle’ for getting through this difficult period in their lives. The majority reported that their social network (family, friends, acquaintances) helped them navigate the complex process by pointing out and directing them to willing physicians and notaries, or by supporting them and the PSAS emotionally and/or by helping with daily tasks.

This reliance on informal networks often stems from a perceived lack of accessible, standardised and empathetic professional support. Rachel, whose son died by AS a few years ago, said:

And so there were several things going on at the same time that influenced each other. For me personally, it was extremely stressful, extremely exhausting. I often cried and felt desperate, and if I hadn't had my partner and my other two children, I don't know what I would have done. They were all really there for me and took care of other things, like going grocery shopping or, I don't know, unloading the dishwasher, feeding the cat. I was also working – not as much as I do now, but still quite a lot. And yes, the stress was just terrible.

(Rachel, Pos.28)

Several interviewees felt the need for psychological support for the PSAS and their family caregivers. While some said that they actively sought help in the form of therapy or self‐help groups, others, particularly the older generation, were resistant to seeking help. Stephanie explained:

And since my mental health had always been a bit up and down, I said last year that I would seek help for myself afterwards. Fortunately, my general practitioner, with whom I can talk very openly, had already asked me at the time if I was open to therapy. So my doctor kind of nudged me in that direction. It was this sense of knowing that if I needed help, it would be there. So I went ahead and found therapy on my own, as well as a psychiatrist who supported me very well last year. I'm also on antidepressants, because I don't think I would have made it through last year otherwise.

(Stephanie, Pos. 115)

Stephanie's account demonstrates the pivotal role of low‐threshold medical interventions in overcoming the barrier to psychological care. Her description of being ‘nudged’ by a trusted physician suggests that for family caregivers, the path to mental health support is often facilitated by pre‐existing professional relationships rather than independent outreach.

Furthermore, her admission that she ‘wouldn't have made it through’ without pharmacological and therapeutic aid underscores the intensity of the psychological strain inherent in the AS process. Stephanie's case illustrates a pattern of delayed self‐care, where the family caregiver prioritises the immediate needs of the PSAS over their own mental stability.

#### 3.1.7 Ramifications in the Post‐Mortem Phase

Even in cases of careful preparation, emotional strain and organisational tasks still continued well after the death occurred. Some interviewees described instances in which professionals reacted emotionally upon learning of the AS (e.g., pharmacists weeping), while others emphasised the substantial bureaucratic demands which extended beyond the dying decree, including the funeral arrangements, estate matters, and post‐death administrative tasks—often made more difficult in the context of AS. Max's account of police involvement in the post‐mortem process provides a detailed and insightful illustration of this issue:

Well, the body was still in the room. And of course, they (*the police*) had to wait until it was picked up. But after they came up with the brilliant idea that it had to go to the forensic medical institute, of course, it wasn't the regular undertakers that came. I had already prepared myself for having to stay there the whole night until someone from the undertakers could fit us into their schedule and collect the body. […] And I wasn't given any further information, although that had been promised. And after quite some time, maybe 14 days, I went to the district commissioner's office to find out what was happening with the body. After all, I also had to organise the funeral.

(Max, Pos. 80, 63)

Many interviewees recounted experiencing a profound sense of shock and disbelief when the anticipated death actually occurred and highlighted the emotional toll resulting from the immediate post‐mortem bureaucracy. Many found the involvement of authorities such as the police, emergency services and medical professionals distressing. The authorities frequently exacerbated the confusion, lacked clear protocols to follow, and were felt by interviewees to lack empathy. Beatrice, a bereaved wife, described how she struggled to determine the next steps in the moments immediately after her husband committed AS:

I made the mistake of Googling what to do when someone dies. And it said to call the police. So I called the police, the emergency number, and explained the situation. And they said, ‘Actually, you need a doctor’. I said, ‘Yes, of course, sorry, I called the wrong number. I'll call a doctor now’. And 2 min later, the police were at the door, of course. Along with paramedics and first responders. I immediately ran out with the dying decree and advanced directive. The first responder immediately knew there was nothing more to be done. […] The first responder informed them (the police; concerning the existence of the Dying Decree Law). Then they consulted with each other. They stayed for a while but discussed the situation, and after half an hour, they left, saying that everything was resolved.

(Beatrice, Pos. 70‐72)

Even though death was anticipated and (in several cases) planned to the day, interviewees said they experienced the post‐mortem proceedings, organisation, and bureaucracy as overwhelming, especially when compounded by emotional grief. Some families found solace in planning together in advance with the PSAS to ensure a smooth process. It is important to note that advance planning gave pre‐mortem solace but did not always prevent chaotic situations from arising when managing the death or its immediate aftermath.

Giselle, a bereaved wife and nurse, recounted that she used her professional knowledge and had help from her professional network to plan and arrange the assisted dying process together with her husband:

[They] had already informed the doctor in advance that the lethal preparation would be taken at 10 a.m. and that we would likely call at around 11 a.m. Later, I just called the funeral home, and they notified the doctor. He arrived right away. I think it was harder for him than for me because he got out of the car, looked at me, recognised me, and knew he was coming to an assisted suicide. His biggest concern was that I might not be able to handle it emotionally […] But I had everything printed out and prepared for him, detailing what he needed to do. At one point, he even received a phone call and said, ‘No, I don't need anything else; the widow has already prepared everything’.

(Giselle, Pos. 108)

### 3.2 Overarching Pattern Across Themes

A cross‐cutting pattern emerged from all seven themes: we found that family caregivers prioritised the PSAS's wishes and needs, often placing them before their own, and only realising in the post‐mortem phase the extent of their own stress and exhaustion. As illustrated by Giselle, the stress of ensuring the process *‘*went right*’* manifested physically only after the burden of responsibility subsided:

The last day was actually the worst day for me, the day before. The day itself, when he died, how should I put it, I'm the kind of person who can manage as long as I have something to do. And as long as I have a plan of tasks to work through, I can cope. But the day before and the evening before, that was terrible. I didn't even realise how much stress I was under. It only became clear afterwards, when I broke out in a rash all over my body. I looked as if I had a contagious disease. That was probably when the stress subsided, because my husband's biggest fear, of course, was that something would go wrong and he would end up as an invalid after all.

(Giselle, Pos. 90)

## 4 Discussion

This study aimed to explore and highlight the invisible labour undertaken by family caregivers throughout the AS process, while revealing the bureaucracy involved in navigating the legal framework. The results of our study provide insight into the experiences of family caregivers of PSAS under Austria's Dying Decree Law. A key aspect common to all identified themes is the lack of information and support structures. We have identified a structural failure to provide accessible information and support structures, leaving family caregivers managing the process of dying by AS in Austria unprepared and alone.

Family caregivers reported feeling distressed by the bureaucratic and legal hurdles they were required to navigate during the AS process in Austria. Furthermore, they were frequently faced with professionals they perceived as opinionated and ill‐educated in the Dying Decree Law, as it is enacted in practice [20]. Our findings align with international research on the pattern of isolation resulting from institutional neglect and/or opposition to introducing policy guidelines on assisted dying [3, 20]. While legislation on assisted dying is specific to each country, there are global patterns with respect to the implementation and practicability of assisted dying laws, reflecting the political priorities in each case.

A study conducted in Switzerland, where there is no legal framework in place and assisted dying is mainly organised by right‐to‐die associations, also showed these patterns of isolation: family caregivers reported receiving little institutional guidance, and discussions were kept to a small circle of people [5]. While the Swiss context differs from Austria due to its reliance on private associations rather than state legislation, the outcome for family caregivers is identical: the Swiss findings align with the support needs and structures we have identified in the Austrian context. Family caregivers placed great importance on the ‘inner circle’ with whom they confided when navigating their way through the legal maze. Another report on the implementation of assisted dying legislation and its lived reality in Belgium and Canada concludes that establishing new processes requires time, and in the period immediately after enactment of the legislation, there is little monitoring and support, leaving families with no real‐time procedural guidance [21]. This suggests that even in more liberalised systems, the administrative focus of assisted dying legal and furthermore practical implementation often neglects the ‘process’ of family caregiver support. A study conducted in Australia has shown that there is an imbalance of power between the institutional stance on assisted dying and patients' ability to choose within the respective legal framework [3]. These international findings also align with the reports of the family caregivers in our study: there is little monitoring and procedural guidance for AS in Austria, leaving those impacted with no support and at risk of isolation. Despite these legal differences, a persistent similarity emerges: the burden of navigating the process falls upon the ‘inner circle’. This suggests that legislation alone does not solve the problem of caregiver burden if it is not accompanied by proactive support pathways.

Our findings on social exclusion and stigmatisation echo both sociocultural and political dimensions of governance in Austria. Despite the legal separation of church and state, catholic dogma continues to shape public policy, especially on matters of life and death. The debate on legalising AS was notably framed by religious arguments, and records show that the final wording of the Dying Decree Law was shaped by dialogue with, among others, ecclesiastical stakeholders [22]. For participants, this structural religious influence did not manifest as a uniform set of personal beliefs, but rather as a pervasive cultural backdrop that reinforced the normative silence surrounding AS. While participants did not explicitly cite religious doctrine, many experienced a climate of social judgment, oftentimes leading to a reluctance to disclose the cause of the death. Consequently, although the law secured the constitutional right to autonomy, there is no political commitment to developing dedicated information centres or support networks. While in this case the omission of such a political mandate has been explained by a lack of financial resources (as establishing policies inevitably incurs costs), and the priority in Austria is to expand palliative and hospice care, it also aligns with the continued influence of prevailing sociocultural values [23, 24].

We have found a pattern of continued silence around the topic of AS, with family caregivers and PSAS not necessarily discussing the process and their wishes with people outside their social network. In contrast, communication within the PSAS's trusted inner circle is intense: when expressing the importance of friends and family for support, interviewees often referred to their inner circle. As such, our findings reflect those of a Swiss study which highlights the importance of social networks, both for organisational matters and emotional support [5]. Our interviewees reported feeling sufficiently emotionally equipped to deal with the situation as they had a social network to rely on but said that they still needed support in navigating the process. The wish for transparent and clear structural guidance provided by an independent but state‐funded institution was voiced by every interviewee.

For all interviewees, open communication played a significant role. Several participants who were partnered or married to the PSAS reported having had discussions about dying by AS with their partners years before the onset of sickness. This voicing of a longing for autonomy at the end of life, even before the biological process of dying commenced, has also been identified elsewhere. Studies have shown that the longing for autonomy to end one's own life can precede the biological trajectory of disease, and that partners routinely share their wishes years in advance. This open communication has helped partners accept the desire to control and hasten death [25, 26].

The international findings on communication throughout the process of assisted dying align with our results: open communications, both in advance and throughout the process of planning death, have helped family caregivers cope with the grief and ensured no unanswered questions remain after the bereavement [7, 25, 26]. Three family caregivers shared that underage children were directly affected by the loss (one parental figure was a PSAS). In two cases, interviewees reported that the children were well informed and that they and the PSAS communicated openly within the inner circle, enabling the children to articulate their perspectives and emotions. In the third case, the AS was unannounced and shielded from the family and excluded all family caregivers from the process.

We looked for patterns regarding age, relationship type, or illness trajectory, but the decisions to include or exclude family members were very diverse. These choices appeared to be driven more by personal family dynamics and the patient's own need for privacy or protection. It is important to note that these insights reflect the caregivers' perspectives on the patient's motives. This highlights how complex and varied family experiences can be when a relative seeks AS.

There are distinct personal differences in dealing with grief pre‐ and post‐mortem, with some people keeping to themselves and others more in need of support. Yet there are also similarities. Family caregivers appear to share the common trait of altruistic behaviour in caring for loved ones who wish to die. A Swiss study has concluded that the process of organising AS involves several dilemmas, and that a key motivation helping family caregivers to overcome various hurdles was to preserve and honour the PSAS's wishes [27]. This also reflects the patterns of altruistic behaviour in family caregivers we have seen in our study: family caregivers often go out of their way and beyond their personal limits to care for their loved one, leaving them exhausted after all the bureaucratic and organisational issues have been resolved post‐mortem.

As seen in other countries, providing PSAS and family caregivers with public guidelines and information material is crucial to ensuring equitable and accessible care at the end of life. Currently, in Austria, no guidelines are available for health professionals and other professional groups on interacting with family caregivers and providing pre‐ and post‐mortem support. The findings presented here underscore the need for concrete operational efforts in this field rather than more research: findings on this topic are consistent across the international landscape.

Following the statements provided by all interviewees, we suggest that this step of action could be delegated to advocacy groups for PSAS and family caregivers. In countries such as Austria, where assisted dying legislation has been enacted but to date no political mandate to provide the necessary support structures has been issued, PSAS and family caregiver advocacy groups could serve as the bridge between the legislation and the public. Advocacy groups consider themselves political actors tasked with representing the needs and interests of affected individuals vis‐à‐vis policymakers [9, 28, 29, 30]. In regard to AS, advocacy groups should raise awareness of the experiences of PSAS and family caregivers within the legal bureaucracy.

To move beyond awareness, these groups should implement concrete support mechanisms, such as standardised ‘procedural navigators’—trained peers or professionals who guide family caregivers through multi‐step legal processes in real‐time. Additionally, advocacy groups could develop structured post‐procedure debriefing programs specifically for family caregivers, addressing the gap in emotional aftercare that currently exists. By issuing clear and strong appeals to policymakers for the public funding of these specific services, advocacy groups could help initiate and establish institutionalised guidance and support. Such institutionalisation would ideally include the creation of a centralised, multilingual, accessible portal and information centre to mitigate the isolation reported by our participants.

### 4.1 Strengths and Limitations

This is the first Austrian study to explore the experiences of family caregivers in navigating the newly enacted Dying Decree Law and addresses a major gap in the understanding of its practicability and implementation. The findings are also pertinent to ongoing debates on assisted dying legislation in other jurisdictions.

As data on established dying decrees and performed AS is scarce in Austria, another strength of this study is that it shares the lived experience of nine different family caregivers, with each case highlighting specific barriers or challenges. Given that there have been fewer than 1000 cases of AS since the law's enactment, securing interviews with nine diverse family caregivers is a notable achievement. However, the recruitment strategy may have introduced selection bias; physicians with more favourable attitudes towards AS may have been more willing to inform families, potentially increasing the likelihood that supportive relatives were overrepresented. Consequently, our sample lacks representation from family caregivers who opposed the PSAS's decision, potentially overrepresenting supportive family caregivers. While we included participants with complex views (e.g., participant A08‐ Naomi), those with particularly negative experiences or strained relationships may have been less likely to be reached or to participate.

The sample was also predominantly female, which may have influenced the findings. Caregiving roles are often gendered, and women's experiences may differ from men's. As a result, the themes identified may primarily reflect female caregiving perspectives. The limited inclusion of male caregivers restricts the transferability of the findings to more gender‐balanced samples.

Furthermore, we must acknowledge the potential recall bias due to the variable time between death and interview (1 month to 3 years), as well as social desirability bias given the sensitive and legally complex nature of AS in Austria.

As the scope of this paper was limited, we were unable to consider palliative care in this context. However, we wish to acknowledge and emphasise the importance of embedding AS within an accessible, high‐quality palliative care system.

Our results may serve to inform policy actors and jurisdictions in the process of reviewing or establishing frameworks for assisted dying, but the findings are grounded in Austria's social and healthcare setting and should therefore be interpreted with caution in other national contexts.

## 5 Conclusion

This study highlights the importance of providing guidance and support to the family caregivers of PSAS. Our findings show that in Austria, family caregivers have to navigate a complex legal maze of bureaucratic requirements, while simultaneously fulfilling the dual role of advocate and caregiver. This study contributes to health policy and caregiving literature by identifying how the absence of institutional support structures effectively shifts the responsibility of legal implementation onto the family, creating a unique form of bureaucratic burden. The global research landscape on AS is diverse, yet a consistent picture emerges: despite substantial differences in legislation, national systems as described in the literature frequently fail to provide adequate and equitable support for PSAS and their family caregivers. As political mandates need time to be introduced after legislation is enacted, we suggest advocacy groups for PSAS and family caregivers should take on the role of raising awareness and working towards the official provision of more institutional guidance and support.

Future research should include longitudinal studies following family caregivers through the bereavement process to assess long‐term impacts. Additionally, comparative studies across different assisted dying frameworks could identify more supportive policy models, while specific research into the experiences of male caregivers would help address the gender imbalance in current data.

## Author Contributions

**Tamina‐Laetitia Vielgrader:** conceptualisation, data curation, formal analysis, investigation, methodology, project administration, resources, software, validation, visualisation, writing – original draft, writing – review and editing. **Ricarda Mewes:** methodology, supervision, writing – review and editing. **Julia Fischer:** validation, writing – review and editing. **Jana Marica Hluch:** resources, formal analysis. **Maria Kletecka‐Pulker:** supervision, project administration. **Gudrun Kreye:** methodology, writing – review and editing. **Elisabeth Lucia Zeilinger:** methodology, supervision, writing – review and editing. The authors acknowledge the significant contributions of all co‐authors and collaborators in the successful completion of this work.

## Ethics Statement

Ethics approval was granted by the University of Vienna Ethics Committee on 11 October 2024, as an addendum to our project ‘Multiperspective evaluation of the Dying Decree Law’ (no 01071).

## Conflicts of Interest

The authors declare no conflicts of interest.

## Supporting information

Supplement 1_ Participant Context.

## Data Availability

The interview guides used in the study will be made available on OSF under https://osf.io/bgpsa upon the study's publication. Additional data cannot be shared due to confidentiality agreements made with study participants in compliance with the study's ethical approval. Any inquiries regarding this matter should be directed to the corresponding author.
