# Supplementary material for: ‘It Simply Required Far Too Many Steps and Made You Feel You Were Just a Number’. Family Caregivers' Experiences With Assisted Suicide in Austria: A Qualitative Study
Source: Health Expect. 2026 Mar 27;29(2):e70651. doi: 10.1111/hex.70651 (PMC13125716; doi:10.1111/hex.70651)
Supplement: Supplementary file 1 — Supplement 1_ Participant Context. [file HEX-29-e70651-s001.docx]

| **Participant** | **Interviewed** | **Context** |
| --- | --- | --- |
| **Max**  A01 | November, 2024 | In an interview, Max, a nearly 80-year-old who volunteers for humanitarian causes, recounts accompanying an acquaintance through the process of assisted suicide. The woman, who was almost blind and did not want to end up like her blind relatives, decided on assisted suicide after watching a television report about euthanasia in Switzerland. Max supported her by organizing the necessary steps in accordance with Austria's Dying Decree Law, including finding notaries and doctors willing to assist with the process.  The woman's desire to die was clear and unwavering, and she was determined to follow through. Max describes the preparation process, the procurement of the medication, and its administration in her own apartment, which she had temporarily left the nursing home to use for this purpose. The situation escalated when a religiously motivated friend of the deceased alerted the police, who arrived shortly after the lethal supplement was taken and attempted to resuscitate the already dying woman, despite the existence of a valid advance directive and dying decree.  Max sharply criticizes the actions of the emergency responders, accusing them of ignoring the law and the dying decree by making unnecessary resuscitation attempts. He points out that the Dying Decree Law lacks binding instructions for emergency services and police, which can lead to such problematic situations. Following the incident, Max reached out to various organizations to highlight the shortcomings of the law and the misconduct of the officers, hoping for improvements in the legal framework.  He emphasizes the need for a centralized point of contact, ideally within the Medical Association, to provide comprehensive information and support for individuals seeking a dying decree, especially for those who are immobile or lack accompaniment. Max advocates for a more liberal and well-thought-out law that ensures the wishes of those affected are respected and that emergency responders are properly trained. |
| **Beatrice**  A02 | December, 2024 | Beatrice recounts the story of her husband’s assisted suicide. He suffered from cancer with metastases and did not want to go to the hospital. He chose this path after the law permitting assisted suicide came into effect. A urologist friend connected him with a doctor who conducted the consultation and reviewed his medical records.  The process, which took about two weeks, included medical assessments and the drafting of a dying decree by a notary. Beatrice emphasized that she wanted to accompany her husband through this step—as they had always done everything together—and that he was already in severe pain. Her husband had always been self-determined; if this option had not existed, he would likely have found another way to avoid hospitalization.  Obtaining the medication from the pharmacy was a significant effort for him, but he insisted on receiving it himself. The family—including their sons and the family dog—was present when he took the lethal supplement in his workshop at home. Beatrice felt a sense of relief when her husband passed away peacefully, as he was finally free from his pain.  After his death, an unexpected situation arose with the police because, in her panic, Beatrice called emergency services instead of contacting a physician. She did not have a general practitioner and did not think to reach out to her husband’s physician. The couple’s friends and family were aware of his intentions and showed great acceptance and support.  Looking back, Beatrice wishes for a centralized point of contact for doctors, notaries, and pharmacies that support assisted suicide, to make the process easier for those affected and their families. She also criticizes the costs involved, which can be a barrier for people with low incomes. The family found comfort in her husband’s self-determination and in facing this difficult time together. |
| **Anna**  A03 | December 2024 | Anna shares that the father of her son, James, passed away last year. Although they had not been a couple for a long time, the family became important again during his severe illness. Anna mentions her 15 years of work as the press officer at an Austrian healthcare institution, which gave her insights into the healthcare system. She learned about the study through her friend Dr. Smith, who supports people with severe illnesses and was aware of James's case and his decision for an assisted death.  James, in his mid-50s, was diagnosed with cancer in December 2022 and passed away in June 2023. Anna found out about his serious illness and poor prognosis in January 2023. For James, it quickly became clear that he would choose assisted suicide. During the remaining six months, his focus was on settling matters, reconciling, and sharing meaningful experiences with his son Louis and friends. Anna and Louis supported him intensively. James was disciplined and brave, even though he struggled with deciding on the exact timing of the assisted suicide. Anna chose not to know the specific date to avoid additional emotional strain.  A central issue was finding a suitable location for the assisted suicide, as hospitals and hospices refused to accommodate it. James spent his final days living with a friend, which Anna found inhumane, as it forces sick individuals to search for shelter. She emphasizes the need for a central point of contact and a dedicated place for assisted suicide. Anna acted as a pragmatic confidante for James, helping him with organizational tasks and supporting him in daily life without crying or complaining. She was grateful that she and Louis could use this intense time to discuss everything and say their goodbyes.  Anna was supported and coached by her friend Dr. Smith, a professional end-of-life companion, which helped her better understand the situation. Her most lasting memories include James's elegance until the very end and the inhumane search for a place to die. She calls on lawmakers to address the issue of finding a location for assisted suicide and to establish clear regulations for the return of unused medications, as many questions remain unanswered regarding the data and handling of these substances. |
| **Rachel**  A04 | January, 2025 | The interviewee, Rachel, a clinical psychologist, reports on the wish to die and the assisted suicide of her 21-year-old son, Alexander, who suffered from a chronical illness since birth and passed away two years ago. She only learned about it when the palliative care physician, Dr. Taylor, appeared at her door. Alexander had found Alexander him through a notary list. Dr. Taylor accompanied Alexander very conscientiously, empathetically, and lovingly, whereas a second doctor provided the required signature very quickly and superficially.  Rachel describes the last two years as a nightmare, marked by Alexander’s progressive physical decline, pain, and dependence on strong medication. A turning point was the death of a friend, which emotionally affected Alexander deeply. The assisted suicide process was lengthy and emotionally taxing, but also characterized by open communication within the family. Alexander had consciously confronted his own death and even prepared something special for his funeral.  Challenges in the process included the high costs (around €12,000 for the notary, doctors, pharmacy, and funeral), the lack of support for people without a social network or financial means, and the stigmatization of the topic. Rachel emphasizes the importance of professional support, such as that provided by Dr. Taylor and a grief counselor, since friends alone are insufficient. She criticizes the unprofessional behavior of a pharmacist when dispensing the medication.  Rachel wishes for a simplification of the process, less stigmatization, and financial support so that money does not become a barrier. Looking back, she describes the time as intense and instructive, as trivial matters lost importance and she experienced a profound sense of connection. She stresses the importance of not being alone during such a difficult period. |
| **Stephanie**  A05 |  | Stephanie, 31, reports on the death of her father, who passed away last year after a terminal diagnosis. Her father, who never wanted to become dependent on care, applied for a dying decree shortly before his death. Although he had always wanted to die autonomously, he had not previously had a living will. The family, particularly Stephanie and her mother, supported his wish, while other relatives struggled to accept it.  The process of obtaining the dying decree was bureaucratic and exhausting. A friend recommended a patient lawyer office, which she described as very helpful. Finding two doctors to provide the required assessments proved difficult, as the trips were very taxing for her father, and one doctor was perceived as unprofessional. The second doctor, however, was very supportive and personable. The standard waiting period for the directive was shortened due to her father’s rapid health decline.  Stephanie emphasizes the enormous bureaucratic burden and lack of empathy in medical institutions when dealing with dying patients. She criticizes that the process often serves more to protect doctors than to support the patient’s well-being. Her father changed his residence in order to apply for the dying decree. However, he died before he could collect the lethal supplement.  Stephanie highlights the importance of psychological support for relatives, who are often left to cope alone in such situations. Stephanie herself received support from her general practitioner and friends, while her mother initially resisted therapy. Organizing matters after her father’s death at home was also challenging but was eased by the assistance of a relative and the funeral service.  In conclusion, Stephanie wishes for better infrastructure for assisted suicides from lawmakers, including central points of contact and more services in underserved areas. She also calls for more comprehensive support for bereaved family members, both regarding bureaucratic matters and psychological processing. She describes the entire process as expensive, lengthy, and full of obstacles, turning dying into a “bureaucratic monster.” |
| **Giselle**  A06 |  | Giselle shares her experiences with her husband’s assisted suicide; he passed away in mid-2023. The counseling process began the year before, shortly after the relevant law came into effect. Her husband, who had been diagnosed with a serious progressive condition, did not want to witness the loss of his independence.  Giselle describes the open communication within the family regarding her husband’s wish to die, noting that he even planned aspects of his own farewell. She emphasizes the importance of him not dying alone and the family having the opportunity to say goodbye over an extended period. The decision for assisted suicide was made to allow for a self-determined and dignified end, rather than experiencing a painful decline.  She criticizes the current legal framework, particularly the waiting period and the exclusion of people with certain cognitive or mental health conditions from assisted suicide. Giselle advocates for reform that would allow people with previously expressed wishes to carry them out, and calls for a state-funded, accessible service to remove current financial and bureaucratic barriers.  The interviewee highlights that her professional background in healthcare helped her approach the situation rationally and obtain the necessary information. She received strong support from her social network and colleagues. Since her husband’s death, Giselle has received numerous inquiries from individuals seeking guidance regarding assisted suicide, underscoring the lack of accessible information and the need for broader public education. |
| **Jane**  A07 |  | The interviewee, Jane, shares her experiences regarding her husband, who has a terminal illness and only has a few weeks left to live. Due to their prior knowledge of assisted suicide laws abroad and their belief that no one should suffer unnecessarily, they decided on assisted suicide in their country of residence, Austria.  The path to the assisted suicide process began about a year and a half ago with her husband’s diagnosis. After a long period of misdiagnoses and treatments that were merely life-prolonging, they decided against further therapy. Jane describes her husband’s physical and psychological suffering, noting that despite severe pain and physical limitations, he clings to life. She emphasizes the importance of the possibility of assisted suicide as an “emergency exit” and a “relief.”  The organization of assisted suicide, including contacting doctors and notaries, was carried out mainly through professional care teams and their own research. Jane highlights that, as a practical and organized person, she is able to manage the emotional burden well in order to support her husband. However, she wishes for improvements in the process, particularly better psychological support for relatives, coverage of costs by health insurance, and safer handling of the lethal medication.  She criticizes the bureaucratic hurdles and the lack of support for those who do not have the financial means or organizational skills. Jane suggests that there should be a dedicated professional group to guide people through this process, similar to a coordinator or planner. She emphasizes that the decision for assisted suicide should be a personal choice, and that in a modern society it is unacceptable to let people suffer while animals are allowed to be euthanized. |
| **Naomi**  A08 |  | Naomi reports on the assisted suicide of her ex-husband in 2022, of which she and the family were unaware. He suffered from a serious health condition that weakened him but did not leave him bedridden. The ex-husband lived in another region, while Naomi and their son lived elsewhere, following a shared custody arrangement. The son, then around twelve years old, could potentially have found his father, as he was discovered several days after the assisted suicide, which Naomi considers unacceptable.  The ex-husband had planned the suicide very deliberately and in an organized manner, including a will and farewell letters, which, however, provided little comfort to the relatives. Naomi interprets the delay in discovery as a spiritually motivated period, allowing time for the soul to depart. She perceived her ex-husband’s suicide as self-determined and dignified for him, but criticizes that the law does not consider the family and leaves them to deal with the consequences alone.  The deceased’s family took over the arrangements after his death. Support services were provided to the family, assisting them over a period of time. Later, the son participated in a children’s support group, which helps children process the loss of a parent to suicide. Naomi wishes that a trusted person were involved in the assisted suicide process to act as a “translator” for the relatives and to prevent situations like delayed discovery.  She criticizes the lack of regulations regarding the discovery of the deceased and the insufficient support for those dispensing the lethal supplement. She compares the situation to other models (like in Switzerland), which she believes have clearer guidelines and would require a support person to be present and authorities to be informed immediately. Naomi found the study to be a safe space to discuss this sensitive topic, which is often taboo in society. |
| **Eloise**  A09 |  | Eloise, a social worker professionally involved with end-of-life care, shares her personal experience with her husband's severe illness and eventual death. Her husband, a highly educated historian, suffered from an aggressive ENT tumor that led to significant disfigurement and daily impairment, including difficulty breathing and eating. Despite extensive palliative care, his condition deteriorated, prompting him to research assisted suicide as an option three months before his death in December 2023.  The process of arranging assisted suicide was fraught with difficulties. Eloise's husband, who could only communicate in writing due to his illness, expressed his wish to pursue a Dying Decree. Eloise fully supported his decision, emphasizing that only the person experiencing such suffering can make this choice. She highlighted the lack of transparency and support for families navigating this process in Austria, particularly regarding finding doctors and pharmacies willing to participate.  Eloise recounted the arduous task of finding two doctors for the necessary assessments, which involved a challenging visit to a second doctor's inaccessible practice. She also faced significant hurdles in locating a pharmacy that would prepare the required medication, eventually finding one that believed they were the only ones offering this service at the time. The process was further complicated by the timing around the Christmas holidays.  Ultimately, her husband passed away on Christmas in a hospice, one day before a scheduled notary appointment for the final step of the Dying Decree. Eloise reflected on the immense burden this process placed on her, balancing work, family, and her husband's care. She expressed a strong desire for greater transparency and support from legal and medical institutions to ease the burden on affected individuals and their families, questioning whether assisted suicide might have offered her husband a more peaceful end than palliative care. |
